# Whole-Genome Sequence and Assembly of Eight Africa Horse Sickness Virus Strains Collected in Namibia and South Africa

**DOI:** 10.1128/mra.01034-22

**Published:** 2023-03-15

**Authors:** Anna Serroni, Sara Traini, Mariangela Iorio, Iolanda Mangone, Luigina Di Gialleonardo, Umberto Molini, Siegfried Khaiseb, Maria Teresa Mercante, Mauro Di Ventura, Marco Caporale

**Affiliations:** a Istituto Zooprofilattico Sperimentale dell’Abruzzo e Molise “G. Caporale,” Teramo, Italy; b School of Veterinary Medicine, Faculty of Health Sciences and Veterinary Medicine, University of Namibia, Neudamm Campus, Windhoek, Namibia; c Central Veterinary Laboratory, Windhoek, Namibia; Portland State University

## Abstract

In this report, we describe eight complete genome sequences of African horse sickness virus (AHSV) strains belonging to four different serotypes, namely, AHSV-5, AHSV-6, AHSV-8, and AHSV-9. Samples were collected in Namibia and South Africa from infected horses between 2000 and 2011. As expected, phylogenetic analyses of the variable outer capsid protein VP2 genomic sequences of AHSV-6 and AHSV-8 show higher nucleotide identity between the isolated viruses than that of the relevant reference strains. The full-genome sequence of AHSV will provide useful information on its geographical origin, and it will also be instrumental for comparing the distribution of the Namibian isolate with that of global isolates.

## ANNOUNCEMENT

African horse sickness (AHS) is a major arthropod-borne disease affecting all Equidae species and is caused by the African horse sickness virus (AHSV) belonging to the *Orbivirus* genus within the *Sedoreoviridae* family (1–3). AHSV is a nonenveloped virus structured into two concentric protein shells surrounding the genome of 10 double-stranded RNA (dsRNA) linear segments designated from segment 1 (S1) to S10 carrying 7 structural proteins (VP1 to VP7) and 5 nonstructural proteins (NS1 to NS4 and NS3A) (3–6).

In this work, blood samples were collected from animals suspected of infection during the febrile stage of disease or from infected organs of dead animals at necropsy (Table 1). Virus isolation was performed on Kenyon Culicoides cell (KC) lines and was subsequently amplified on BSR cells (a clone of baby hamster kidney-21 cells) (7, 8). Viral dsRNA was extracted as described previously from cell lysates (9). Full-length amplification of cDNAs was performed with the full-length amplification of cDNA (FLAC) method followed by reverse transcription-PCR (RT-PCR) (10). Amplified DNA was subsequently purified using DNA clean and concentrator 5 kit according to the manufacturer’s instructions (Zymo Research) and was quantified with the Qubit 2.0 fluorometer (Thermo Fisher Scientific).

**TABLE 1:** Description of eight AHSV genome sequences obtained from samples collected from infected horses in Namibia and South Africa

Virus protein	Data by virus name					
	AHSV-5 Swanepoel South Africa (lung)^*a*^			AHSV-6 Okahandja Namibia (spleen)^*b*^		
	No. of mapped reads (%)	Final coverage (×)	GenBank accession no.	No. of mapped reads (%)	Final coverage (×)	GenBank accession no.
VP1	125,286 (10.91%)	6,581	OP455575	87,499 (11.12)	5,660	OP455585
VP2	267,479 (23.29)	7,244	OP455576	178,028 (22.63)	7,131	OP455586
VP3	196,754 (17.13)	7,067	OP455577	103,004 (13.09)	6,544	OP455587
VP4	20,041 (1.75)	2,930	OP455578	66,190 (8.41)	6,520	OP455588
NS1	57,759 (5.03)	6,890	OP455579	28,460 (3.62)	4,791	OP455589
VP5	99,598 (8.67)	6,614	OP455580	76,416 (9.71)	6,758	OP455590
VP7	42,505 (3.70)	6,117	OP455581	28,473 (3.62)	4,873	OP455591
NS2	112,004 (9.75)	6,387	OP455582	15,838 (2.01)	3,964	OP455592
VP6	21,479 (1.87)	4,657	OP455583	39,599 (5.03)	5,948	OP455593
NS3	32,005 (2.79)	5,647	OP455584	10,465 (1.33)	3,378	OP455594
	AHSV-6 Omaruru Namibia (blood)^*c*^			AHSV-9 Okahandja Namibia (blood)^*d*^		
	No. of mapped reads (%)	Final coverage (×)	GenBank accession no.	No. of mapped reads (%)	Final coverage (×)	GenBank accession no.
VP1	6,746 (0.46)	560	OP508188	5,847 (0.46)	481	OP508198
VP2	12,376 (0.85)	1,252	OP508189	3,809 (0.30)	416	OP508199
VP3	39,382 (2.70)	4,555	OP508190	17,905 (1.42)	2,081	OP508200
VP4	45,338 (3.11)	5,702	OP508191	34,884 (2.76)	5,191	OP508201
NS1	251,453 (17.26)	7,584	OP508192	94,529 (7.49)	7,284	OP508202
VP5	124,283 (8.53)	7,535	OP508193	41,519 (3.29)	6,385	OP508203
VP7	9,902 (0.68)	2,705	OP508194	194,471 (15.41)	7,404	OP508204
NS2	138,409 (9.50)	7,362	OP508195	174,371 (13.81)	7,383	OP508205
VP6	282,733 (19.41)	7,486	OP508196	14,653 (1.16)	3,868	OP508206
NS3	155,235 (10.66)	7,670	OP508197	69,841 (5.53)	4,833	OP508207
	AHSV-8 Windhoek/A Namibia (lung)^*e*^			AHSV-8 Windhoek/B Namibia (lung)^*f*^		
	No. of mapped reads (%)	Final coverage (×)	GenBank accession no.	No. of mapped reads (%)	Final coverage (×)	GenBank accession no.
VP1	62,747 (13.11)	4,924	OP432769	115,763 (8.81)	6,440	OP455595
VP2	86,772 (18.13)	6,606	OP432770	119,618 (9.10)	7,077	OP455596
VP3	59,395 (12.41)	5,244	OP432771	230,920 (17.57)	7,246	OP455597
VP4	26,266 (5.49)	4,170	OP432772	46,609 (3.55)	5,894	OP455598
NS1	35,886 (7.50)	5,509	OP432773	86,966 (6.62)	7,295	OP455599
VP5	35,661 (7.45)	6,026	OP432774	77,256 (5.88)	6,905	OP455600
VP7	15,852 (3.31)	4,292	OP432775	97,321 (7.40)	7,214	OP455601
NS2	32,426 (6.78)	5,843	OP432776	140,240 (10.67)	7,209	OP455602
VP6	3,932 (0.82)	1,072	OP432777	16,559 (1.26)	4,003	OP455603
NS3	7,621 (1.59)	3,731	OP432778	18,616 (1.42)	5,125	OP455604
	AHSV-6 Gobabis Namibia (liver)^*g*^			AHSV-8 Windhoek/C Namibia (spleen)^*h*^		
	No. of mapped reads (%)	Final coverage (×)	GenBank accession no.	No. of mapped reads (%)	Final coverage (×)	GenBank accession no.
VP1	7,829 (4.15)	624	OP508208	35,274 (12.04)	2,787	OP455605
VP2	17,533 (9.30)	1,723	OP508209	29,628 (10.11)	2,878	OP455606
VP3	18,603 (9.87)	2,106	OP508210	24,507 (8.36)	2,764	OP455607
VP4	5,320 (2.82)	851	OP508211	13,954 (4.76)	2,237	OP455608
NS1	8,545 (4.53)	1,600	OP508212	27,355 (9.34)	4,451	OP455609
VP5	9,286 (4.93)	1,871	OP508213	12,260 (4.18)	2,469	OP455610
VP7	2,114 (1.12)	573	OP508214	7,070 (2.41)	1,929	OP455611
NS2	6,984 (3.70)	1,885	OP508215	17,700 (6.04)	4,739	OP455612
VP6	18,809 (9.98)	5,152	OP508216	646 (0.22)	198	OP455613
NS3	4,402 (2.33)	2,150	OP508217	11,510 (3.93)	4,860	OP455614

a Total no. of reads 1,148,307; ID26463205; reference sequences are KM886344 to KM886353. AHSV-5 was kindly provided by Robert Swanepoel.

b Total no. of reads 786,707; ID26463206; reference sequences are KP009741 to KP009750.

c Total no. of reads 1,456,701; ID26463207; reference sequences are KP009741 to KP009750.

d Total no. of reads 1,262,196; ID26463208; reference sequences are KF860036 to KF860045.

e Total no. of reads 478,556; ID26463209; reference sequences are KF860026 to KF860035.

f Total no. of reads 1,314,546; ID26463210; reference sequences are KF860026 to KF860035.

g Total no. of reads 188,537; ID26463211; reference sequences are KP009741 to KP009750.

h Total no. of reads 293,009; ID26463212; reference sequences are KF860026 to KF860035.

Libraries were prepared using Nextera XT library prep kit (Illumina Inc., San Diego, CA) according to the manufacturer’s protocol. Deep sequencing was performed on the NextSeq500 instrument (Illumina Inc.) using the NextSeq 500/550 mid output reagent cartridge v2 (300 cycle) (Illumina Inc.) and standard 150-bp paired-end reads.

Read quality control was performed using fastqc v0.11.5, and the reads were trimmed using trimmomatic v0.36 (11) using the following parameters: ILLUMINACLIP:/trimmomatic-0.36-6/adapters/NexteraPE-PE.fa:2:30:10 LEADING:25 TRAILING:25 SLIDINGWINDOW:20:25 MINLEN:36. The resulting high-quality reads underwent a depletion step to remove the host reads using Bowtie 2.1.0 and the GCF_000349665 *Mesocricetus* host genome as a reference (12). The remaining reads were *de novo* assembled using SPAdes assembler v3.11 (13). In order to identify the best reference sequences, the scaffolds were compared to the nucleotide and nonredundant databases using BLAST (14). Full genomes were obtained by mapping the reads to the identified AHSV reference sequences using Bowtie 2.1.0 (12) and by subsequently using the MegAlign software (v17.2, DNASTAR, Inc., Madison, WI) (15). Sequences were submitted to NCBI and accession numbers within the reference used, the mapped reads, and the coverage obtained are detailed in Table 1 (16). All software tools were run with default parameters unless otherwise specified.

**FIG 1:**
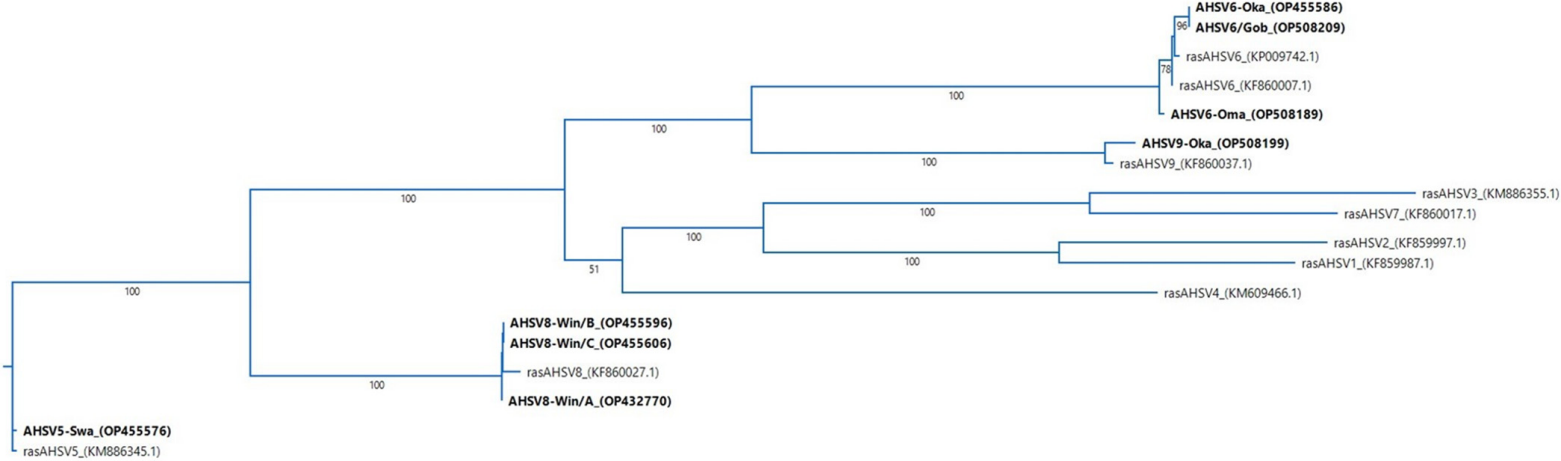
Phylogenetic trees of AHSV genome segment 2. VP2 genomic sequences from the 7 Namibian and 1 South African virus isolates together with the AHSV reference strains were used to generate the phylogenetic tree using maximum likelihood RAxML and bootstrapped values were obtained using 100 replicates. VP2 sequences of this study are in bold. Newly isolated viral sequences of AHSV-6 and AHSV-8 show higher nucleotide identity between circulating serotypes than the relevant reference virus strains.

The phylogenetic tree of VP2 gene nucleotide sequences (segment 2) was elaborated using the maximum likelihood RAxML method and was constructed using full-length genomic sequences in a pair-wise deletion, p-distance algorithm and was bootstrapped using 100 replicates using MegAlign software. The different VP2 genes identifying the different serotypes are indicated in Fig. 1 together with outgroups.

In conclusion, in this work, we describe the sequence analysis of eight AHSVs representing four different serotypes (AHSV-5, AHSV-6, AHSV-8, and AHSV-9), isolated from infected horses during AHS outbreaks which occurred in Namibia and South Africa between 2000 and 2011 (Table 1). The data presented increases the low number of full-length AHSV genomes publicly available and provide useful information on the geographical origin of the circulating AHSV strains.

### Data availability.

The complete genome sequences of the eight AHSV strains are available in GenBank, under accession numbers listed in Table 1. The reference genomes used for the annotation were published previously (16). AHSV sequences raw data were submitted to SRA under BioProject PRJNA930321.
